# Interleukin-15-armored GPC3-CAR T cells for patients with solid cancers

**DOI:** 10.21203/rs.3.rs-4103623/v1

**Published:** 2024-04-03

**Authors:** David Steffin, Nisha Ghatwai, Antonino Montalbano, Purva Rathi, Amy N Courtney, Azlann B Arnett, Julien Fleurence, Ramy Sweidan, Thao Wang, Huimin Zhang, Prakash Masand, John M Maris, Daniel Martinez, Jennifer Pogoriler, Navin Varadarajan, Sachin G Thakkar, Deborah Lyon, Natasha Lapteva, Zhuyong Mei, Kalyani Patel, Dolores Lopez-Terrada, Carlos Ramos, Premal Lulla, Tannaz Armaghany, Bambi J Grilley, Gianpietro Dotti, Leonid S Metelitsa, Helen E Heslop, Malcolm K Brenner, Pavel Sumazin, Andras Heczey

**Affiliations:** 1 Texas Children’s Cancer Center, Department of Pediatrics, Baylor College of Medicine, Houston, Texas.; 2 Center for Advanced Innate Cell Therapy, Baylor College of Medicine, Houston, Texas.; 3 Dan L Duncan Comprehensive Cancer Center, Baylor College of Medicine, Houston, Texas.; 4 Center for Cell and Gene Therapy, Baylor College of Medicine, Houston, Texas.; 5 Department of Immunology and Microbiology, Baylor College of Medicine, Texas.; 6 Department of Radiology, Baylor College of Medicine, Houston, Texas.; 7 Department of Pediatrics, Children’s Hospital of Philadelphia and Perelman School of Medicine at the University of Pennsylvania, Philadelphia, Pennsylvania.; 8 Pathology and Laboratory Medicine, Children’s Hospital of Philadelphia and Perelman School of Medicine at the University of Pennsylvania, Philadelphia, Pennsylvania.; 9 William A. Brookshire Department of Chemical and Biomolecular Engineering, University of Houston, Houston, Texas.; 10 Department of Pathology and Immunology, Baylor College of Medicine, Houston, Texas.; 11 Department of Pathology, Baylor College of Medicine, Houston, Texas.; 12 Lineberger Comprehensive Cancer Center, University of North Carolina, Chapel Hill, North Carolina.; 13 Texas Children’s Hospital Liver Tumor Program, Houston, Texas.

## Abstract

Interleukin-15 (IL15) promotes the survival of T lymphocytes and enhances the antitumor properties of CAR T cells in preclinical models of solid neoplasms in which CAR T cells have limited efficacy^1–4^. Glypican-3 (GPC3) is expressed in a group of solid cancers^5–10^, and here we report the first evaluation in humans of the effects of IL15 co-expression on GPC3-CAR T cells. Cohort 1 patients (NCT02905188/NCT02932956) received GPC3-CAR T cells, which were safe but produced no objective antitumor responses and reached peak expansion at two weeks. Cohort 2 patients (NCT05103631/NCT04377932) received GPC3-CAR T cells that co-expressed IL15 (15.CAR), which mediated significantly increased cell expansion and induced a disease control rate of 66% and antitumor response rate of 33%. Infusion of 15.CAR T cells was associated with increased incidence of cytokine release syndrome, which was rapidly ameliorated by activation of the inducible caspase 9 safety switch. Compared to non-responders, tumor-infiltrating 15.CAR T cells from responders showed repression of SWI/SNF epigenetic regulators and upregulation of FOS and JUN family members as well as genes related to type I interferon signaling. Collectively, these results demonstrate that IL15 increases the expansion, intratumoral survival, and antitumor activity of GPC3-CAR T cells in patients.

## Introduction

Genetically engineered T lymphocytes expressing chimeric antigen receptors (CARs) mediate over 80% complete response rates in patients with relapsed or refractory B cell leukemias^11,12^ and have significant potential to improve the survival of patients with solid neoplasms. Conventional chemo- and radiotherapies have limited ability to eliminate bulky or metastatic solid cancers and are associated with significant short- and long-term toxicities; thus, new and effective therapies are needed. The efficacy of CAR T cells has been limited in patients with solid tumors^13^ in part due to the tumor microenvironment (TME), which contains inhibitory signals that block immune responses and lacks supportive factors including cytokines (i.e. interleukin-15 (IL15)) required for survival and optimal function of tumor-specific T cells^1^. IL15 belongs to the common gamma chain cytokine family and is important for CD8 T cell memory formation, mitochondrial metabolism, and the expansion and persistence of antigen-experienced T cells^2^. In nonclinical models, IL15 co-expression in CAR T cells significantly improves their ability to expand, persist, and induce complete tumor regression^3,4,14^; however, it is unknown how IL15 impacts CAR T cell antitumor activity and safety in humans.

Glypican-3 (GPC3) is expressed in a group of solid neoplasms including hepatocellular carcinoma (HCC), the third most common cause of cancer-related death in the world^5–9^. It is not expressed in non-malignant tissues, making it an attractive immunotherapeutic target^10^. We have previously shown that IL15 co-expression increases the expansion and antitumor activity of GPC3-CAR T cells in nonclinical solid tumor models^15^. To study GPC3-CAR T cells co-expressing IL15 (15.CAR) in humans, we assessed a total of 24 patients, 12 with CAR T cells and 12 with 15.CAR T cells in ongoing Phase 1 studies. Here, we report safety characteristics and antitumor response rates, establish expansion kinetics in the peripheral blood, describe trafficking to tumor tissues, and determine gene expression changes in CAR and 15.CAR T cells in the peripheral blood and within tumors using single-cell RNA sequencing.

### 15.CAR T cell safety

**Fig 1.**
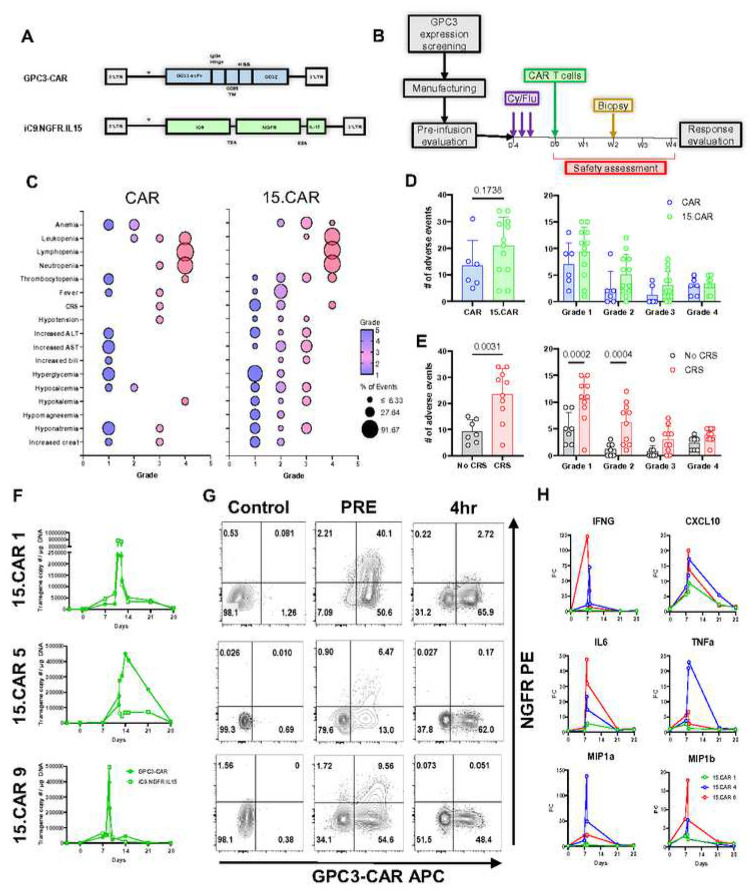
Safety characteristics of CAR and 15.CAR infusions: **A.** Transgene maps of GPC3-CAR and iC9.NGFR.IL15 constructs used to co-transduce T cells to generate infusion products. **B.** Schematic representation of patient enrollment and treatment. **C.** Bubble plots representing frequency of the indicated adverse events for CAR (left) and 15.CAR (right) infused patients. Adverse events (AEs) were collected from Day −4 until Day +28 post-infusion and graded according to the Common Terminology Criteria of AEs v5. Color spectrum corresponds to AE grade 1–5, bubble size corresponds to frequencies. **D.** Comparison of frequency of AEs between CAR vs 15.CAR and **E.** Comparison of AEs between patients with and without CRS using two-tailed T test and two-way ANOVA with Sidac correction, respectively. Mean ± SD. **F-H.** Levels of GPC3-CAR and iC9.NGFR.IL15 expressing T cells quantified by qPCR (F) and flow cytometry (G) and changes in concentrations of indicated serum cytokines (H) in peripheral blood of patients treated with rimiducid, the chemical inducer of the iC9 safety switch.

Immunotherapeutic targeting of GPC3 has been previously established in adults using antibodies, vaccines, and CAR T cells^16–18^. We confirmed the absence of GPC3 expression in children using a comprehensive, non-malignant pediatric tissue array^19^ and as part of eligibility prior to the enrollment of each patient, GPC3 expression in tumor samples was quantified by immunohistochemistry^20^ (**Extended data fig 1A-C)**. Patients were enrolled to receive either CAR T cells or 15.CAR T cells (Fig. 1A, **Extended data fig 2 and Extended data table 1**). All patients underwent lymphodepletion with cyclophosphamide and fludarabine, followed by cell infusion and 28 days of monitoring to assess safety (Fig. 1B). Six patients were infused with CAR T cells on dose level (DL)1 at 1×10^7^ CAR T cells/m^2^, and six were infused on DL2 at 3×10^7^/m^2^ (children on NCT02932956, adults on NCT02905188). On both DLs 1 and 2, the infusions were safe, no antitumor responses were observed, CAR T cells were detected in the peripheral blood and tumor tissues, and no significant differences were found in these measures between DLs (**Extended data fig 3**). Most grade 3–4 toxicities were related to lymphodepletion and are common in patients receiving cell therapy. Next, 12 patients were infused with 15.CAR T cells at 3×10^7^/m^2^ (DL2; children on NCT04377932, adults on NCT05103631). There was not a significant difference in the number of adverse events (AEs) on DL2 in patients treated with CAR (n=6) versus 15.CAR (n=12) T cells (Fig 1C–D). In the CAR group, 1 of 6 patients developed cytokine release syndrome (CRS) that required treatment with at least immunomodulation (IL1 or −6 inhibition) versus 9 of 12 patients in the 15.CAR group (relative risk 3.3, 95% confidence interval 1.226 to 9.723, p=0.043). As expected, AEs were more common in patients with CRS with more grade 1 and 2 AEs were observed in this group, though there was not an increase in number of grade 3 or 4 AEs (Fig 1E). Changes in circulating cytokine levels including IL15 were similar in CAR and 15.CAR cohorts (**Extended data fig 4A, B),** and patients with CRS had increased concentrations of IFNγ, CCL2, TNFα, eotaxin, IL6, CXCL10, MIP1β, and IL15 (**Extended data fig S4C** and **D**). The inducible caspase 9 (iC9) safety switch was deployed in three patients treated with 15.CAR T cells^21^. The first patient (15.CAR 1) had a prolonged grade 3 CRS event with fever, tachycardia, tachypnoea, and required >40% O_2_ via high-flow nasal cannula. The second patient (15.CAR 5) had a prolonged grade 2 event with fever, tachycardia, tachypnoea, and less than 40% O_2_ requirement. The third patient (15.CAR 9), who had a history of smoking and chronic obstructive pulmonary disease (40 packs/year), developed fever, tachycardia, and hypoxia which required mechanical ventilation. All three patients received a single intravenous dose of rimiducid, the chemical inducer of dimerization for iC9, after which all three showed rapid improvement of symptoms, effective reduction of circulating 15.CAR T cells, and normalization of inflammatory cytokine levels (Fig 1F–H). These results demonstrate that CAR T-related toxicities can be quickly resolved if needed in patients who are refractory to IL1/6 inhibition.

### Antitumor responses mediated by 15.CAR T cells.

**Fig 2.**
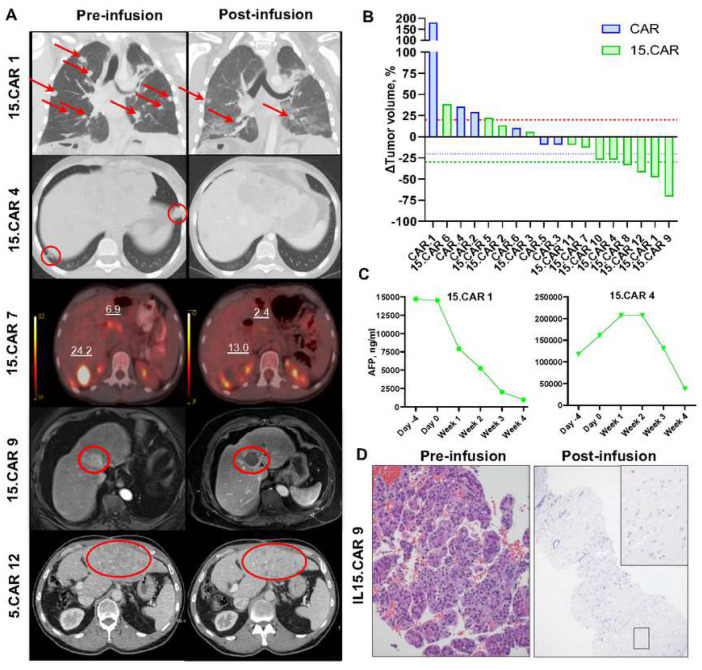
15.GPC3-CAR T cells induce significant antitumor responses in patients. Antitumor responses were determined by comparing pre- and post-infusion 3D imaging. **A.** Coronal CT chest (15.CAR 1), axial CT chest (15.CAR 4), PETCT (15.CAR 7), MRI abdomen (15.CAR 9) and axial CT abdomen (15.CAR 12) based images showing pre- and post-CAR T cell infusion. Red arrows and circles represent tumors. **B.** Waterfall plot representing changes in tumor volumes of patients treated with 3 × 10^7^/m^2^ with CAR or 15.CAR T cells. Red line: 20% increase, Blue line: 20% decrease, Green line: 30% decrease. **C.** Serum alpha-feto protein (AFP) concentrations at indicated timepoints in responders with AFP secreting neoplasms. **D.** Pre- and post-infusion tumor biopsy assessed with hematoxylin-eosin staining showing near complete necrosis of patient 15.CAR 9’s liver tumor.

To compare antitumor response rates in patients infused with CAR versus 15.CAR T cells, we evaluated changes in pre- and post-infusion 3D imaging and serum alpha-feto protein (AFP) concentrations. Objective responses were not detected in the six CAR cohort patients infused at 3 × 10^7^ CAR T cells/m^2^ (DL2); three patients had progressive disease (PD), and three patients had stable disease (SD). In contrast, among the 12 patients infused with 15.CAR T cells on the same dose level, four had PD, four had SD, and four had a partial response (PR) according to RECIST criteria^22^ (**Extended data table 1**). Among those with SD, patients 15.CAR 4 and 15.CAR 10 had a greater than 26% reduction in tumor burden. Patient 15.CAR 7 had an approximately 12.8% reduction and significant decrease in PET avidity of residual masses, providing evidence of antitumor activity even if it did not meet RECIST criteria (Fig 2A, B**; Extended data fig 5A**). Two responding patients had AFP-secreting tumors, and both showed significant reduction of AFP levels (Fig 2C**, Extended data fig 5B**). When comparing serum cytokine profiles, eotaxin and CCL22 concentrations were elevated in responders (**Extended data fig 5C,D**). In 15.CAR 9, an MRI of the patient’s liver tumor suggested complete necrosis, and image-guided sampling of the lesions confirmed near-complete necrosis of the primary liver tumor (Fig 2D). Collectively, patients treated with 15.CAR T cells had a disease control rate (SD and PR) of 66% (8/12) and an objective response rate of 33.3% (4/12).

### Multiomic baseline characteristics and *in vivo* expansion of CAR and 15.CAR T cells

**Fig 3.**
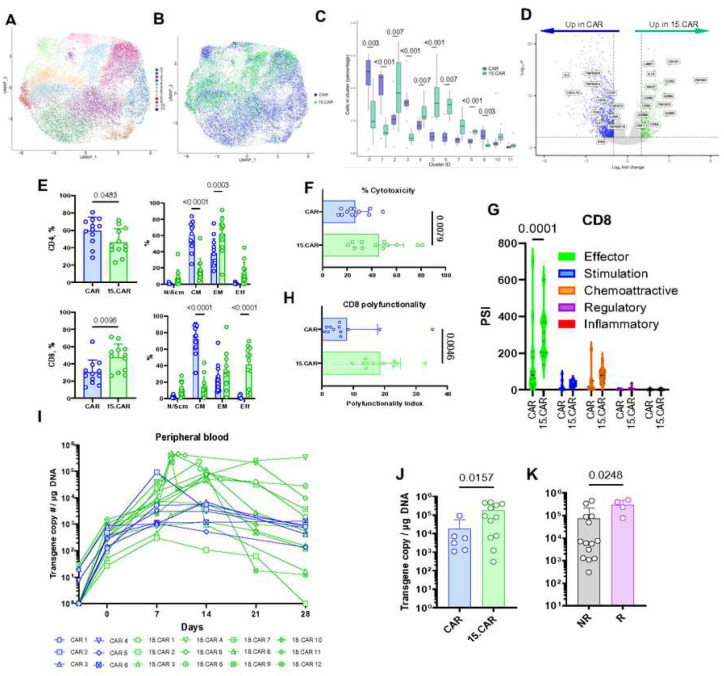
Comparison of pre-infusion products and expansion in patients of CAR and 15.CAR T cells. Products were first assessed with single cell RNA sequencing. **A.** Uniform Manifold Approximation and Projection (UMAP) identifying unique T cell clusters in integrated CAR and 15.CAR pre-infusion products. **B-C.** Differential representation of CAR vs 15.CAR T cells in clusters shown in UMAP projection (**B**) and proportion for each cluster (**C**). Center line: median, box limits: first and third quartiles, whiskers: 1.5x the interquartile range, dots: outliers. **D.** Differentially expressed genes in CAR vs 15.CAR products. **E.** Frequencies of CD4/8 and effector / memory subsets in CAR vs 15.CAR products by flow cytometry. Two-tailed, unpaired T test and two-way ANOVA with Šídák correction, respectively. **F.** Cytotoxicity of CAR vs 15.CAR products measured by ^51^Cr release assay. **G.** Polyfunctionality strength index comparing vs 15.CAR T cell product’s cytokine production by Isoplexis. Two-way ANOVA with Šídák correction. **H.** Differentially expressed cytokines in CD8 subsets of CAR and 15.CAR T cell products. **I.** Peripheral blood CAR T cell frequencies quantified by qPCR at indicated timepoints for each patient. **J.** Comparison of peak expansion on dose level 2 of CAR vs 15.CAR T cells post-infusion. Two-tailed, unpaired T test. Data represented as mean ± SD. **K.** Comparison of expansion of cells in responders vs non-responders and Mann-Whitney test. Data represented as mean ± SD.

Specific transcriptomic and cell surface phenotypic characteristics of CAR T cell infusion products have been associated with differences in clinical outcomes^23,24^. To compare the baseline gene expression profile of CAR and 15.CAR T cells, we analyzed a total of 36,722 cells via single-cell RNA sequencing (scRNAseq). We found that CAR and 15.CAR T cell products had differential enrichment of gene expression across 12 unique cell clusters (C0-C11; Fig 3A–C). We identified 3,285 differentially expressed genes in 15.CAR T versus CAR T cell products including increased expression of *CD8A/B*, *ZNF683* (encoding HOBIT), and genes related to cytolytic activity (i.e. *GZM*s, *PRF1*, *NKG7*) as well as downregulation of costimulatory receptors (*TNFRSF4*, *TNFRSSF9* and *TNFRSF18*) and *TCF7* (Fig 3D; **Extended data fig 6A**). At the protein level, compared to CAR T cell products, 15.CAR T cells were enriched for the CD8 subset and showed a significantly lower frequency of central memory cells with a corresponding increase in effector memory and effector subsets (Fig 3E). Compared to CAR T products, 15.CAR products had a similar frequency of PD1+ and TIM3+ cells but higher frequency of LAG3+ cells. Double negative CD39/CD69 cells, which are associated with improved antitumor responses in patients treated with tumor-infiltrating lymphocytes^25^, had similar frequency in both products (**Extended data fig 6B**). As T cells differentiate from naïve into memory and then terminal effector cells, their ability to kill and produce cytokines increases. Consistent with gene expression and phenotype results, 15.CAR T cells showed significantly higher cytolytic activity and were more polyfunctional^26^ than CAR T cells, primarily due to producing more effector cytokines (Fig 3F–G, **Extended data fig 6C,D**). Sustained antigen-dependent proliferation is necessary for CAR T cells to maintain or increase the pool of tumor-specific effectors and reduce tumor masses with large numbers of neoplastic cells. 15.CAR T cells expanded significantly more than CAR T cells in the peripheral blood, and this difference was also significant in responders versus non-responders in the 15.CAR cohort (Fig 3 I–K; **Extended data fig 7A-C**). The frequency of tumor-infiltrating CAR and 15.CAR T cells was similar; however, these results may have been biased in some patients infused with 15.CAR T cells due to activation of the iC9 safety switch and resultant reduction in cell numbers at the time of biopsy (**Extended data fig 7D, E**). Collectively, these results show that 15.CAR T cell products were more poised pre-infusion to execute effector function and were able to expand better in patients than CAR T cells. These factors likely contributed to overall more potent antitumor activity.

### Gene expression evolution of 15.CAR T cells in peripheral blood and tumors.

**Fig 4.**
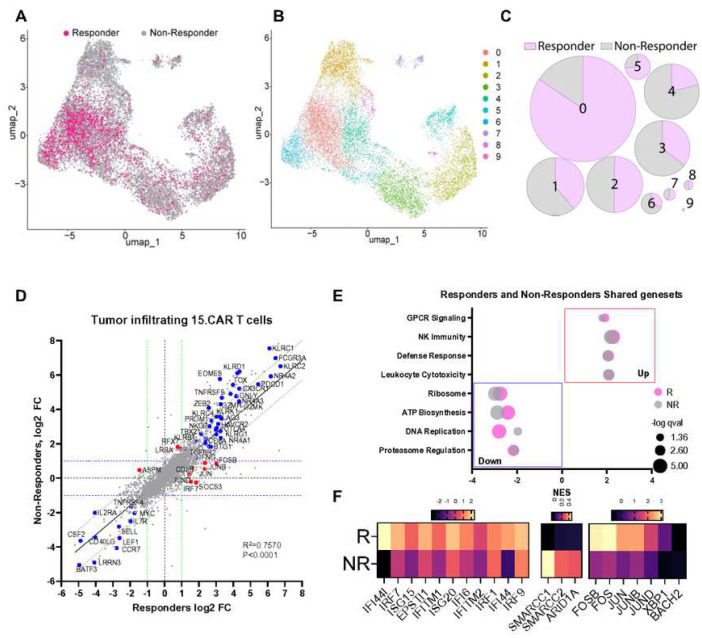
Comparing the single cell gene expression profile of tumor infiltrating 15.CAR T cells post-infusion in responders vs non-responders. The transcriptomic profile of Infusion products and tumor infiltrating 15.CAR T cells were interrogated with single cell RNA sequencing. Differentially expressed genes (DEGs) for indicated groups were determined by comparing the product- with peripheral blood tumor-derived 15.CAR T cells. **A.** UMAP projection of tumor infiltrating 15.CAR T cells from responders and non-responders. **B.** Unsupervised clustering of cells corresponding of tumor infiltrating CAR T cells of responders and non-responders from the 15.CAR cohort. **C.** Differences in cluster proportions for indicated groups. **D.** DEG comparison (product vs tumor infiltrating 15.CAR T cells) in responders (x axis) vs non-responders(y axis) from the 15.CAR cohort. **E.** Gene sets enriched in tumor infiltrating 15.CAR T cells. **F.** Heatmap representing the genes with most differences in change from baseline in responders vs non-responders in 15.CAR T cells.

The transcriptomic evolution of CAR T cells in the peripheral blood and the TME of patients with solid tumors is poorly understood, and gene expression profiles associated with increased expansion and superior antitumor responses of infused CAR T cells remain to be established. We used scRNAseq to compare the gene expression profiles of pre-infusion products with cells collected from the peripheral blood (35,906 cells) and tumor biopsies (10,382 cells) at 2–3 weeks post-infusion. Both CAR and 15.CAR T cells collected from the peripheral blood demonstrated upregulation of genes and gene sets associated with NK-like differentiation, cytotoxic effector activity^27^, and exhaustion^28^. Additionally, genes associated with less differentiated memory cells and cytokine signaling as well as corresponding gene sets (i.e. chromatin remodeling and mitotic spindle organization)^29^ were downregulated in both groups (**Extended data Fig 8A,B**). These observations suggest that both CAR and 15.CAR T cells were exposed to tumor cells and had initiated effector differentiation leading to decreased proliferative capacity. However, the gene expression profiles of CAR T and 15.CAR T cells in the peripheral blood diverged for genes involved in adhesion/effector function, IL15 signaling, cellular metabolism, NFkB signaling, innate antiviral response, and survival (**Extended data Fig 8A and C**)^30,31^. Because T cells captured from the peripheral blood are different from those in tumor tissues, we also evaluated the gene expression profile of adoptively transferred cells isolated from tumors. We could not capture sufficient CAR T cells for scRNAseq analysis from the tumor biopsies of patients in the CAR cohort, and therefore it was not possible to compare CAR and 15.CAR cohorts. In contrast, tumor-infiltrating 15.CAR T cells were captured effectively, and we compared gene expression in responders with evidence of antitumor activity (at least >20% reduction of tumor size) versus non-responders. 10 clusters of tumor infiltrating 15.CAR T cells were identified; responders were enriched in Cluster 0 and 5 while cells from non-responders dominated Clusters 4, 7 and 8. Cells from responders showed upregulation of genes related to T cell activation, memory formation, Type I IFN signaling (T1IFN) and AP1 family members (Fig 4A–C, **Extended data Fig 8D**). To determine the evolution of 15.CAR T cells post-infusion, we compared their gene expression change to baseline (pre-infusion product). For both responders and non-responders, genes associated with cytotoxicity, NK-like transition, terminal effector differentiation, and exhaustion were upregulated. In parallel, genes related to less differentiated naïve and memory subsets were downregulated in both groups (Fig 4D). Gene ontology analyses showed that both responders and non-responders were enriched for expression of programs related to membrane-initiated signaling, NK immunity, cytotoxic response, and downregulation of programs related to ATP generation and replication (Fig 4E, **Supplementary table 2)** demonstrating activation and effector differentiation in the TME while losing proliferative capacity. However, in contrast to cells from non-responders, 15.CAR T cells from responders showed upregulation of AP1 family members *FOS*, *FOSB, JUN, JUNB*, and *JUND*, regulators of T cell survival, and genes associated with T1IFN signaling as well as repression of genes in the SWI/SNF chromosome remodeling complex^32,33^ (Fig 4F). These changes demonstrate robust evolution in the TME and identify genes and programs associated with responses in patients treated with 15.CAR T cells.

## Discussion:

CAR T cells induce significant antitumor responses in patients with hematologic malignancies and have tremendous promise to help patients with solid tumors. Recent clinical studies have demonstrated higher frequencies of antitumor responses in patients with low-burden neuroblastoma^34^ and brain tumors treated with repeat infusions^35^.

In this first-in-human assessment, we evaluated the impact of transgenic IL15 expression in GPC3-CAR T cells in patients with GPC3-positive solid cancers. Systemic administration of IL15 has been associated with significant toxicities due to high serum concentrations^36^. In this study, while CRS was more common in 15.CAR- versus CAR T-treated patients, IL15 serum concentrations were not higher, suggesting that these events were likely due to pronounced T cell activation. CRS-related side effects were effectively controlled through IL6/IL1 blockade in most patients. The iC9 safety switch has been proven to effectively eliminate alloreactive T cells in patients^37^. Here, we provide the first evidence that iC9 mediates rapid elimination of CRS induced by CAR T cells that is resistant to other interventions.

Antitumor responses were more frequent in patients treated with 15.CAR T cells compared to patients treated with CAR T cells and to patients treated on a previous phase 1 study of T cells expressing a third-generation GPC3-CAR^17^. The antitumor activity of 15.CAR T cells may be underestimated in our study as we have not yet evaluated higher doses as dose escalation is ongoing in our Phase 1 trial and repeat infusion schedules may also be considered.

Previous preclinical reports raised concerns about malignant transformation of T cells engineered with IL15^38^. Our study provides evidence for absence of IL15-mediated transformation in T cells manufactured from mature peripheral blood mononuclear cells (PBMCs). In fact, T cells expressing the iC9.IL15 construct without the GPC3-CAR were not detectable shortly after infusion, suggesting that *in vivo* expansion of these cells remains antigen-dependent. Unexpectedly, elimination of iC9.15-expressing cells reduced populations of both CAR-positive and CAR/IL15-dual positive circulating populations, raising the possibility that paracrine- or cross-presentation of IL15 promotes CAR T cell survival. Identifying the specific dose of rimiducid to eliminate side effects without significant effect on CAR T cell persistence is a focus of active investigation in our group.

IL15 maintains CD8 T cells and promotes memory formation^2^.15.CAR T cells contained a higher proportion of CD8+ cells but showed a more effector differentiated transcriptomic and cell surface phenotype with higher *ex vivo* cytolytic activity and increase in polyfunctionality. Since the products were manufactured under identical conditions including supplementation with IL7 and IL15^39^, the co-transduction procedure may have contributed to these differences.

Understanding the evolution of gene expression programs in CAR T cells post-infusion should provide critical insights to identify master regulator genes and gene sets associated with cell survival and sustained effector function. CAR T cells co-expressing *JUN* had increased antitumor activity in nonclinical models^40^, and our results demonstrate the importance of FOS and JUN subfamily members, as these genes were significantly upregulated in tumor-infiltrating 15.CAR T cells in responders. Previously, *ex vivo* screening methods demonstrated that decreasing the function of the SWI/SNF chromosomal remodeling complex can enhance CAR T cell antitumor function^33^. Consistent with this observation, we found that *ARID1A* expression was repressed in tumor-infiltrating 15.CAR T cells of responders. *IRF7* expression has been shown to be necessary for CAR T cell survival^41^, but results from nonclinical models have been inconclusive as to whether IRF7 is associated with exhaustion or antitumor function^41–43^. Our results from tumor-infiltrating 15.CAR T cells provide evidence that *IRF7* and associated factors related to T1IFN signaling indeed play a role in supporting antitumor function in the human TME. To establish whether these gene expression changes are drivers of or are simply associated with clinical responses will be a focus of future *ex vivo* gain- and loss-of-function experiments.

In conclusion, single infusions of 15.CAR T cells are safe and mediate increased antitumor response rates and expansion in patients with solid tumors. Further, increased expression of *FOS/JUN* and T1IFN pathway-related genes as well as downregulation of SWI/SNF activity in intratumoral 15.CAR T cells were associated with response to therapy.

## Methods:

### Clinical Study Design and regulatory approvals:

We conducted four phase 1 clinical trials evaluating autologous T cells expressing a second-generation, GPC3-specific chimeric antigen receptor incorporating the 41BB costimulatory endodomain^44^ and two of which also co-expressing the cytokine interleukin-15 (IL-15) to treat pediatric (GAP; NCT02932956, AGAR; NCT04377932) and adult (GLYCAR; NCT02905188, CATCH NCT05103631) patients with relapsed and/or refractory liver tumors. All patients were included in safety analysis. All trials were registered at clinicaltrials.gov prior to the start of enrollment. All patients received lymphodepletion with cyclophosphamide (500mg/m^2^/dose) and fludarabine (30mg/m^2^/dose) on days −4, −3, and −2 followed by the infusion of GPC3-CAR T cells on Day 0 - +2 on two dose levels (dose level 1 (DL1) at 1×10^7^ and DL2 at 3×10^7^ CAR+ T cells/m^2^). Dose escalation followed the standard 3+3 design for the GPC3-CAR T cell trials, and the Bayesian Optimal Interval (BOIN) design for 15.GPC3-CAR T cell trials. Study objectives: The primary aim i) determine the safety of escalating doses of GPC3-CAR T cells, and ii) to determine the recommended phase 2 dose (RP2D) of GPC3-CAR T cells in treating patients with GPC3-positive solid tumors after lymphodepleting chemotherapy. Secondary objectives were to i) assess the anti-tumor effect of the infused GPC3-specific CAR T cells in patients with GPC3-positive solid tumors, and ii) to assess the in vivo persistence, phenotype and functional activity of infused GPC3-CAR T cells in children with GPC3-positive solid tumors. History and physical exam, along with laboratory testing was performed on Day −4, day 0, and at weeks 1,2, and 4 post infusion for all patients. Adverse events (AEs) were collected from the start of lymphodepletion (Day −4) until Day 28 post-infusion and described according to the Common Terminology Criteria for Adverse Events, version 5 (CTCAEv5). Dose-limiting toxicities were defined as any of the following that may be considered at least possibly related to the study cellular products: 1) Any Grade 5 event; 2) Non-hematologic dose-limiting toxicity is any Grade 3 or Grade 4 non-hematologic toxicity that fails to return to Grade 2 within 72 hours; 3) Grade 2 to 4 allergic reaction to CAR T cell infusion; 4) Grade 4 hematologic toxicity that persists for 28 days or greater; 5) Grade 3 cytokine release syndrome (CRS) infusion reactions and neurologic toxicity if they fail to return to Grade 1 within 7 days; 6) Grade 4 CRS and neurologic toxicities. Clinical response assessment: Standard 3D imaging using computed tomography (CT) of the chest and CT or magnetic resonance imaging (MRI) of the abdomen were performed within 2 weeks prior to GPC3-CAR T cell infusion and again at 4 weeks (range 4–8 weeks). Antitumor response rate was defined by RECIST criteria as previously described^22^.

### Patient Eligibility and Logistics:

The studies were conducted in two phases: Procurement and Treatment.

#### Procurement eligibility:

1) Relapsed or refractory GPC3-positive solid tumors; 2) Age >1 year and ≤18 years; 3) Lansky or Karnofsky score >60%; 3) Life expectancy >16 weeks; 4) Child-Pugh-Turcotte score <7 (for patients with hepatocellular carcinoma only; 5) Informed consent explained to, understood by and signed by patient/guardian.

#### Exclusion criteria for procurement:

1) History of hypersensitivity reactions to murine protein-containing products OR presence of human anti-mouse antibody (HAMA) prior to enrollment (only patients who have received prior therapy with murine antibodies); 2) History of organ transplantation; 3) Known HIV positivity; 4) Severe previous toxicity from cyclophosphamide or fludarabine.

#### Treatment eligibility:

In addition to criteria included for procurement: 1) Barcelona Clinic Liver Cancer Stage A, B or C (for patients with hepatocellular carcinoma only); 3) Life expectancy of ≥ 12 weeks; 2) Child-Pugh-Turcotte score < 7 (for patients with hepatocellular carcinoma only); 3) Creatinine clearance as estimated by Cockcroft Gault or Schwartz ≥ 60 ml/min; 4) serum AST< 5 times the upper limit of normal (ULN); 5) total bilirubin < 3 times ULN for age; 6) INR ≤1.7 (for patients with hepatocellular carcinoma only); 7) absolute neutrophil count > 500/μl; 8) platelet count > 25,000/μl (can be transfused); 9) hemoglobin ≥ 7.0 g/dl (can be transfused); 10) pulse oximetry >90% on room air; 11) refractory or relapsed disease after treatment with up-front therapy and at least one salvage treatment cycle; 12) recovery from acute toxic effects of all prior chemotherapy and investigational agents before entering this study; 13) birth control for 3 months after the T-cell infusion in sexually active patients;

#### Exclusion criteria for treatment:

In addition to exclusion criteria included for procurement: 1) Pregnancy or lactation; 2) uncontrolled infection 3) Systemic steroid treatment (0.5 mg prednisone equivalent/kg/day); 4) Known HIV positivity; 5) Active bacterial, fungal or viral infection (except Hepatitis B or Hepatitis C virus infections).

The clinical trials and corresponding protocols were reviewed and approved by the Protocol Review Committee, the Institutional Biosafety Committee and the Institutional Review Board at Baylor College of Medicine and the US Food and Drug Administration (FDA). Children were enrolled at the Texas Children’s Hospital and adults were treated at Houston Methodist Hospital by members for the Center for Cell and Gene Therapy of Baylor College of Medicine, Houston, Texas, in accordance with Declaration of Helsinki principles. All participants and/or legal guardians provided written informed consent / assent prior to enrollment on the studies.

Patients with at least stable disease were eligible for reinfusion if meeting all treatment eligibility criteria. Patients DL1.CAR3, 15.CAR4, 15.CAR7, 15.CAR8 and 15.CAR12 received a second infusion.

### Clinical grade vector production:

Both vectors (GPC3.CAR.41BBζ and iC9.NGFR.IL15) included a standard replication incompetent retrovirus produced from the PG13 packaging producer cell line, which provides gag/pol and GALV env in trans. The vector genome for each were derived from the SFG backbone, a Moloney-based splicing retroviral vector, which lacks all coding for env and most of the gag-pol gene except for the packaging sequence. The vector was further modified by introducing base pair substitutions at position 413, 430 and 635 of the Mo-MuLV sequence to prevent translation of any portion of the remaining gag sequence. For GPC.CAR.41BBζ a codon-optimized mini gene was synthesized by GeneArt^®^ (Thermo Fisher Scientific, Waltham, MA) encoding a human immunoglobulin heavy-chain leader peptide and the GPC3-specific single chain variable fragment (scFv) GC33. The mini gene was subcloned in frame into a retroviral vector containing an expression cassette encoding an IgG1 short hinge, a CD28 transmembrane domain (CD28TM), and 41BB.ζ signaling domains^44^. The transgene integration was confirmed with sequencing, and the producer cell clone was validated under Good Manufacturing Practice guidelines. The final viral supplement was stored at −80°C and tested prior to release for clinical testing. For iC9.NGFR.IL15, A codon-optimized mini gene was synthesized by GeneArt^®^ including one T2A-like sequence encoding a 20 amino acid-peptide from Thosea Asigna insect virus (RAEGRGSLLTCGDVEENPGP) and one E2A-like sequence encoding a 20 amino acid-peptide from Equine rhinitis A virus (RAQCTNYALLKLAGDVESNPGP). These connect inducible caspase 9 (iC9), the truncated NGFR gene coding the extracellular and transmembrane domain, and the human interleukin-15 gene. The expression cassette was cloned into the SFG retroviral vector backbone.

### CAR T cell manufacturing:

GPC3-CAR T cells for both the protocols were generated using peripheral blood mononuclear cells (PBMCs) from patients first stimulated with CD3 and CD28 (Miltenyi Biotec) mAbs in the presence of recombinant human IL-7 (10 ng/ml) and IL-15 (5 ng/mL, both from R&D Systems) on day 1 and transduced with retroviral particles encoding the GPC3-CAR construct in 24-well, RetroNectin-coated plates (Takara Bio) on day 3. Next, T cells were washed and replated on day 5, expanded and tested followed by cryopreservation on Day 8. 15.GPC3-CAR T cells were also generated using PBMCs stimulated with CD3 and CD28 mAbs in the presence of recombinant human IL-7 and IL-15 on day 1. They were transduced with retroviral particles encoding the iC9.NGFR.IL15 construct in RetroNectin-coated plates on day 3, resuspended and transduced with retroviral particles encoding the GPC3-CAR construct on day 4.

### Immunophenotyping of GPC3-CAR T cell products and post infusion, peripheral blood samples:

GPC3- and 15.GPC3-CAR T cell products and peripheral blood samples were assessed with flow cytometry using BUV395-Conjugated Mouse Anti-Human CD4 (Clone RPA-T4, BD Biosciences), BUV496-Conjugated Mouse Anti-Human CD8 (Clone RPA-T8, BD Biosciences), BUV 737-Conjugated Mouse Anti-Human TIM-3 (Clone 7D3, BD Biosciences), BV421-Conjugated Mouse Anti-Human CD25 (Clone M-A251, BD Biosciences), BV480-Conjugated Mouse Anti-Human CD45RO (Clone UCHL1, BD Biosciences), BV650-conjugated Mouse Anti-Human CD279 (Clone MIH4, BD Biosciences), BV711-conjugated Mouse-Anti-Human CD69 (Clone FN50, BD Biosciences), BV786-Conjugated Mouse Anti-Human LAG3 (Clone FN50, BD Biosciences), BV605-Conjugated Mouse Anti-Human CD3 (Clone SK7, BD Biosciences), APC-R700-Conjugated Mouse Anti-Human CD127 (Clone HIL-7R-M21,BD Biosciences), FITC-Conjugated Mouse Anti-Human CD197 (CCR7) (Clone 150503, BD Biosciences), BD Pharmingen^™^ PE-Conjugated Mouse Anti-Human CD271 (Clone C40–1457, BD Biosciences), PerCP-Cy^™^5.5-Conjugated Mouse Anti-Human CD39 (Clone TU66, BD Biosciences) and Viability Stain 780.

Transduction efficiency of peripheral blood samples was assessed in products and peripheral blood via the following antibodies: GPC3-CAR expression was measured by Alexa Fluor^®^ 647-Conjugated AffiniPure Goat Anti-Mouse IgG, F(ab’)2 fragment specific (Polyclonal, Jackson Immunoresearch) and IL15 was measured by FITC-Conjugated Mouse Anti-Human NGFR (C40–1457, BD Biosciences). Nonspecific binding was mediated using Monoclonal Anti-Bovine IgG antibody (BG18, Sigma Aldrich).

For pre-infusion CAR T cell products and post-infusion peripheral blood mononuclear cells (PBMCs), the lymphocyte region was selected on forward and side scatter, followed by hierarchical gating focused on populations of interest.

### Cytotoxicity assessment:

2 × 10^6^ target cells were labeled with 0.1 mCi (3.7MBq) ^51^Cr and mixed with effector cells at the following effector to target ratios: 1) 40:1; 20:1; 10:1; 5:1. Target cells were incubated in Click’s Medium with 5% glutamine and 1% FBS. To determine maximum ^51^Cr release 5×10^4^/ml target cells are separately incubated in Triton X-100. Target cells and effector cells were incubated for four hours, at which point supernatants were collected and radioactivity was measured in a gamma counter (PerkinElmer; Wellesley; MA). The mean percentage of specific lysis of triplicate wells was calculated according to the following formula: (test release–spontaneous release)/(maximal release–spontaneous release) × 100.

### Single-cell cytokine production quantification:

Cryopreserved CAR and 15.CAR T cell products were thawed in RPMI 1640 medium (Fisher, MT10040CV) supplemented with 10% FBS (Sigma, F2442-6X500 mL), 1x Glutamax (Thermo,35050061). Cells were then recovered overnight in complete RPMI medium with recombinant human IL-7 (10 ng/ml) and IL-15 (5 ng/mL) from R&D Systems at a density of 1 × 10^6^ cells/mL in a 37°C, 5% CO_2_ incubator. Cells were stimulated with HUH-7 tumor cell line at a 1:1 ratio of 1 × 10^6^ cells/mL for each for 24 hours at 37°C, 5% CO2. CD4^+^/CD8^+^ T-cell subsets were then separated using anti-CD4 or anti-CD8 microbeads (Miltenyi, 130-045-101/130-045-201). The stimulated cells were labeled with membrane stain (1:500 dilution, IsoPlexis), resuspended in complete RPMI medium at a density of 1 × 10^6^ cells/mL and cells were loaded into a human adaptive IsoCode Chip (IsoPlexis). Cells on the chip were incubated at 37°C, 5% CO2 for additional 13.5 hours on IsoLight automation system (IsoPlexis). Polyfunctionality of T cells defined as a cell co-secreting 2+ cytokines were analyzed by the IsoSpeak software across seven functional groups: Th1 Pro-inflammatory (GM-CSF, IFN-γ, IL-2, IL-12, TNF-α, TNF-β); Th2 Pro-inflammatory (IL-4, IL-5, IL-7, IL-9, IL-13); Chemoattractive (CCL11, IL-8, IP-10, MCP-1, MCP-4, MIP1-α, MIP-1β, RANTES); Regulatory (IL-10, IL-15, IL-22, TGF-β1); Th17 Pro-inflammatory (IL-1β, IL-6, IL-17A, IL-17F, IL-21); Cytolytic (Granzyme B, perforin); and Other (sCD40L, sCD137). The polyfunctional strength Index (PSI) of T cells was computed using a pre-specified formula, defined as the percentage of polyfunctional cells, multiplied by the sum of the mean fluorescence intensity (MFI) of the proteins secreted by those cells. The functional groups of T cells were deconvoluted and visualized by 3D t-Distributed Stochastic Neighbor Embedding (3D-tSNE) and heatmap visualizations.

### Quantification of CAR T cells with quantitative PCR:

Evaluation of CAR T cell and NGFR-IL15 persistence was assessed by calculating the copy number of either the CAR or iC9.NGFR.IL15 transgene after extracting genomic DNA from PBMCs with QIAamp DNA Blood Minikit (QIAGEN) according to the manufacturer’s manual and measuring the transgene copy number with RT-PCR using primers (forward: 5′-AGCTGCCGATTTCCAGAAGA −3′; reverse: 3′- GCGCTCCTGCTGAACTTCA −5′) and probe (5′-AAGGAGGATGTGAACTGAGA-3’) sequences (Applied Biosystems) in the ABI Prism 7700 Sequence Detector (PerkinElmer). The copy number was normalized to 1 μg of DNA of PBMCs in the patient samples and transgene copy number was normalized per milliliter of patient peripheral blood at all time points.

### Quantification of serum Cytokine levels:

Serum cytokine levels were measured with the Milliplex MAP magnetic-bead-based multi-analyte panel (EMD Millipore) on the Luminex 200 system (Luminex) with the xPONENT (Luminex) software according to the manufacturer’s manual.

### Rimiducid supply and dosing:

Rimiducid is the chemical inducer of dimerization and activates the iC9 safety switch. Rimiducid was manufactured and provided by Bellicum Pharmaceuticals. The rimiducid was diluted in normal saline with volume as appropriate for weight and administered via IV infusion at the target dose. Rimiducid was given at weight adjusted doses between 0.004–0.03 mg/kg/dose. Patients only received a single dose in this study.”

### Single-cell RNA sequencing and processing.

Cells were dissociated, and cell libraries were prepared according to Chromium Next GEM Single Cell 5’ Reagent Kit v2 kit (10x Genomics). Samples had >95% viability. Cells were labeled using a 10x Genomics Chromium Controller and full-length cDNA was synthesized, barcoded, and amplified by PCR. KAPA Library Quantification kit (Roche) was used to quantify libraries. Libraries were sequenced with NovaSeq 6000 (Illumina) to a sequencing depth of ~500 million reads. The GPC3-CAR sequence has a murine-derived GPC3-specific single-chain variable fragment and an MMLV packaging signal. The GPC3-CAR sequence was indexed together with the human reference genome (version: GRCh38.p13) to generate a chimeric reference before data pre-processing and read alignment. Alignment to the chimeric reference followed the 10x Genomics standard, using Cell Ranger (v7.1.0) and tertiary analysis with Seurat tools. Cells with raw UMI > 0 matching the GPC3-CAR sequence were identified as CAR+ cells. Before cell clustering, quality controls were conducted in Seurat^39^ (v4.0). Genes detected in less than 10 cells, cells with fewer than 200 profiled genes, and more than 10% of mitochondrial Raw unique molecular identifiers (UMIs) were removed to exclude low-quality or dying cells. Cells with more than 7,000 genes were excluded. Doublets were identified using DoubletFinder. Because of the sparsity of CAR T cells—particularly in the CAR cohort—spike-in-primers were employed in post-library preparation to increase the detection limit of CAR T cells in scRNA-Seq assay with limited CAR T representations. The following sequences were used to amplify the GPC3-CAR transgene; 5’:GATCTACACTCTTTCCCTACACGACGC, 3’:TAAACTTCTGGGAATATGCTGTATCCCCGGTTT. After cleanup, enzymatic fragmentation and size selection were used to generate variable-length fragments that collectively span the entire transcript. Library construction was carried out via End repair, A-tailing, Adaptor ligation, and PCR amplification. UMI counts were Log Normalized with a scale factor of 10k. The most 2,000 variable marker genes were retained and scaled (linear transformation) for clustering and integration. CAR+ cells in pre-infusion product samples were integrated using harmony^45^. When clustering CAR T product samples, we performed principal component analysis (PCA) on the scaled data and unsupervised Louvain clustering analysis with a resolution of 0.5. A total of 12 clusters were identified from 24 pre-infusion product samples. Cells in each cluster were projected onto a two-dimensional UMAP. Where available, CAR+ cells from pre-infusion product-, peripheral blood-, and tumor biopsy-derived data were integrated using harmony. B cells, red blood cells, and monocytes were filtered out by removing cells where HBB, CD79A, MS4A1, CD19, CD22, CD14, and MS4A7 reads were > 0.

### Differential gene and gene-set expression analysis.

Differential gene expression in product vs. peripheral blood in the IL15.CAR vs. CAR evolution comparison was performed as follows. We randomly downsampled each donor down to 125 single cells. Raw read counts from each donor were then summed from all the cells by gene using the Seurat function *PseudobulkExpression(pb.method = “aggregate”)*. Differential gene expression between product and peripheral blood samples was calculated using DESeq2^46^ for IL15.CAR and CAR separately. The DESeq2 parameters used for these analyses were *DESeq(test=“LRT”, sfType=“poscounts”, useT=T, reduced=~1)*. Shrinkage of effect size was calculated using the DESeq2 function *lfcShrink(type=apeglm, svalue=T)*. We performed this resampling procedure 200 times, values were then averaged to produce p-value estimates. Fold change estimates were produced by comparing psuedobulk profiles across CAR Ts in each sample, using a psuedocount of 1. For cluster proportion statistical analysis, 2-tailed p values were calculated using a hypergeometric distribution comparing the composition of each cluster--to responder vs. non-responder cells--and the composition of the total cell population. Differential gene expression in product vs. biopsy in the Responder vs. Non-Responder evolution comparison was performed as described above with the following differences: cells from each donor were randomly down-sampled to match the size of the cohort with fewest identified CAR T cells. Gene Ontology gene sets with FDR <0.05 for up- or down enrichment were identified using geneset enrichment analysis. Differential gene expression in tumor Responders vs. Non-Responders was performed using the Seurat function FindMarkers(test.use = “wilcox_limma”, min.pct = 0.3, min.diff.pct = 0.1).

### Statistical Analysis:

All patients were included in primary and safety analyses. Descriptive statistics were used to describe phenotypic data and T cell expansion. Plots of growth curves demonstrating measurements over time within patients were generated to visualize patterns of immune reconstitution. Comparisons were made between groups using Wilcoxon rank-sum test or t test, whichever was appropriate, for continuous variables and the Fisher exact test for categorical variables. Changes from baseline to follow-up measures were compared using the Wilcoxon signed rank test. Confidence intervals for relative risk were calculated with Koopman asymptomatic score and p value was estimated by Fisher’s exact test. Statistics were computed using GraphPad Prism 10 (GraphPad Software). Differences were considered significant when p < 0.05.

## Data Availability

Data will be available at GEO under accession number GSE253352.
